# Effect of Elicitation with Iron Chelate and Sodium Metasilicate on Phenolic Compounds in Legume Sprouts

**DOI:** 10.3390/molecules26051345

**Published:** 2021-03-03

**Authors:** Henryk Dębski, Wiesław Wiczkowski, Marcin Horbowicz

**Affiliations:** 1 Faculty of Exact and Natural Sciences, Institute of Biological Sciences, Siedlce University of Natural Sciences and Humanities, Prusa 14, 08-110 Siedlce, Poland; henryk.debski@uph.edu.pl; 2 Department of Chemistry and Biodynamics of Food, Institute of Animal Reproduction and Food Research of the Polish Academy of Sciences, Tuwima 10, 10-748 Olsztyn, Poland; w.wiczkowski@pan.olsztyn.pl

**Keywords:** fenugreek, lentil, alfalfa, sprouts, elicitation, flavonoid, phenolic acid, iron, silicon

## Abstract

Seven-day-old sprouts of fenugreek (*Trigonella foenum-graecum* L.), lentil (*Lens culinaris* L.), and alfalfa (*Medicago* *sativa* L.) were studied. The legume seeds and then sprouts were soaked each day for 30 min during 6 days with water (control) or mixture of Fe-EDTA and sodium silicate (Optysil), or sodium silicate (Na-Sil) alone. Germination and sprout growing was carried out at temperature 20 ± 2 °C in 16/8 h (day/night) conditions. Phenolic compounds (free, ester, and glycosides) content were determined by HPLC-ESI-MS/MS using a multiple reaction monitoring of selected ions. Flavonoids and phenolic acids were released from their esters after acid hydrolysis and from glycosides by alkaline hydrolysis. The presence and high content of (−)-epicatechin (EC) in fenugreek sprouts was demonstrated for the first time. Applied elicitors decreased the level of free EC in fenugreek and alfalfa sprouts but enhanced the content of its esters. Besides, elicitors decreased the content of quercetin glycosides in lentil and fenugreek sprouts but increased the content of quercetin and apigenin glycosides in alfalfa sprouts. The applied elicitors decreased the glycoside levels of most phenolic acids in lentil and p-hydroxybenzoic acid in fenugreek, while they increased the content of this acid in alfalfa. The mixture of iron chelate and sodium silicate had less effect on changes in flavonoid and phenolic acid content in legume sprouts than silicate alone. In general, the used elicitors increased the content of total phenolic compounds in fenugreek and alfalfa sprouts and decreased the content in lentil sprouts. Among the evaluated elicitors, Optysil seems to be worth recommending due to the presence of iron chelate, which can be used to enrich sprouts with this element.

## 1. Introduction

An alternative method of increasing the nutritional value of food is to germinate seeds in order to obtain sprouts that have more beneficial dietary properties compared to seeds [1,2,3]. Sprouts are recommended by nutritionists due to their high content of bioactive compounds, such as flavonoids, phenolic acids, vitamins, and easily absorbable minerals [4,5]. These compounds play an important role in protecting the human body from various types of chronic disorders such as cardiovascular disease, diabetes, and cancer [6,7]. As a result of the growth of the sprouts for a several days at light conditions, it is possible to obtain young plants called microgreens [8]. Sprouting also decreases the content of antinutrients such as trypsin inhibitors and phytic acid [9].

The contents of secondary plant metabolites are dependent on stressful growth conditions, such as UV-irradiation, drought, high light, nutrient deficiencies, temperature, and herbicide [10]. An alternative method that effectively increases the accumulation of secondary metabolites (including phenolics) in sprouts is elicitation [11,12,13,14,15,16,17]. 

Deficiency of certain elements in people′s diet leads to a search for improvement [18]. Elicitation is also a tool for fortification of food with important minerals [19,20,21]. The use of an elicitor containing iron makes it possible to produce sprouts with an increase of this element’s content, which is a valuable feature of such a procedure [3,22].

Legume seeds are generally difficult to digest, so germination before consumption makes the digestion process easier. The most popular species used to produce sprouts are soybean, mung bean, lentils (*Lens culinaris* L.), and alfalfa (*Medicago sativa* L.) [4,5,11,12,13,14]. Less known and consumed are fenugreek (*Trigonella foenum-graecum* L.) sprouts [23]. Fenugreek is an annual herb that grows in the Mediterranean countries and Asia [24]. Its sprouts have been shown to be rich in polyphenols and minerals, allowing the germinated seeds to be included in functional foods [1,24]. An extract obtained from fenugreek seeds has been shown to be cytotoxic in vitro to breast, pancreatic, and prostate cancer cell lines, but not to normal cells [25]. Fenugreek is also produced as powdered herb, capsules, and tablets for nutritional supplement [26]. In traditional medicine, alfalfa sprouts are used for treatment of arthritis, kidney problems, and diabetes, as well as in coronary diseases [26,27,28]. Sprouted lentil contains high quality proteins, fats, and polyphenols, and is also a good source of minerals such as copper and zinc [29,30]. In recent years, numerous reports have been published on the influence of biotic and abiotic elicitors on the quality of lentil sprouts [31,32,33,34]. Most of them concern the influence of the applied elicitors on the content of polyphenolic compounds and antioxidant activity [6,33,35,36,37].

Flavonoids and phenolic acids are a large group of compounds that perform important functions in plants due to their high antioxidant potential, ability to chelate metals, prevention of lipids against oxidation, or activation enzymatic defense system [11,36,37]. Since the 1990s, there has been a growing interest in the health benefits of a diet rich in plant food, in which these compounds are essential ingredients [38]. Epidemiological studies show that products containing flavan-3-ols reduce the risk of cardiovascular diseases [39,40]. Results of another study in healthy adults have also shown the benefits of flavan-3-ols consumption on cardiovascular quality [41,42]. The (−)-epicatechin has higher activity in arterial dilation response than catechin [43,44,45]. Furthermore, high consumption of products containing flavan-3-ols reduced the risk of developing type 2 diabetes, regardless of other aspects of the diet [46]. 

Soluble phenolic acids and flavonoids that occur in plants can be divided into free, esterified, and glycosidic forms [47]. The insoluble-bound phenolics are localized in the cell wall matrix of the plant cells and can be released by fermentation, germination, and/or other food processing methods [48]. Therefore, the extraction procedure used for isolation of the phenolic compounds from plant tissue could have a significant impact on the results of their analysis [49,50,51].

Growth stimulator Optysil is a commercial formulation that contains sodium silicate and iron chelate (Fe-EDTA), which are easily assimilated by plants and thus strengthen cell walls and increase plant tolerance to abiotic stresses [52]. Silicon also increases the resistance of plants to diseases and pests [53,54]. In the available literature, there is no information concerning the influence of this bio-stimulant on legume sprouts. Therefore, the aim of this study was to determine an effect of Optysil and compare it to sodium silicate (Na-Sil) on the accumulation of soluble flavonoids and phenolic acids, both free and their esters and glycosides in seven-day-old fenugreek, lentil, and alfalfa sprouts.

## 2. Results

The identification of phenolic compounds present in the legume sprouts was achieved using the LC-ESI-MS/MS method using a multiple reaction monitoring of selected ions and by comparison of their retention times with authentic standards. The analysis showed the presence of free (‒)-epicatechin (EC), flavones, flavonols, flavonone, and phenolic acids, as well as their esters and glycosides. No *iso*-rhamnetin, syringic, and chlorogenic acid were found in the sprouts of the tested species, but clear differences were found in the composition and content of phenolic compounds. Contents of phenolics differed significantly across species and used elicitors. What draws attention is a particularly high content of EC in fenugreek sprouts. The EC occurred mainly in the esterified forms, probably as epigallocatechin gallate, which represent 70% to 90% of total flavonoids content (Table 1). 

It is worth noting that glycosides of the EC were not found in fenugreek and lentil sprouts, and alfalfa sprouts contained only small amounts of its esters. The mixture of Fe-EDTA and Na-Sil (Fe-EDTA–Na-Sil) applied during germination, and sprout growth resulted in lowering the free EC content and increasing its esters. However, a greater reduction in free EC content in fenugreek sprouts and a further increase in EC ester levels was caused by Na-Sil alone. The increase in the EC esters content under the influence of both elicitors also occurred in alfalfa sprouts. The naringenin content in the sprouts of the evaluated legumes was low and the applied elicitors did not significantly affect its level (Table 1).

In the sprouts of the tested legumes, quercetin and kaempferol derivatives were found (Table 2). In the lentil and alfalfa sprouts, the free and derivatives of these flavonols were the major flavonoids. The content of its glycosides decreased under the influence of the Na-Sil and Fe-EDTA–Na-Sil in fenugreek and lentil sprouts. Na-Sil increased the glycoside levels of both quercetin and kaempferol in alfalfa sprouts. 

Apart from flavan-3-ols and flavonols, sprouts of some of the species tested contained measurable levels of apigenin and luteolin and their C-glycosides vitexin and orientin (Table 3). No measurable apigenin and vitexin content was found in lentil sprouts, as well as vitexin in alfalfa. In fenugreek, the level of apigenin was relatively low, and the used elicitors did not affect all its forms. However, in alfalfa sprouts the content of free apigenin was significantly reduced, and its glycosides increased under the influence of both elicitors. The use of Na-Sil alone caused much greater changes in the content of these flavones than the use of Fe-EDTA–Na-Sil. In fenugreek and lentil sprouts, the level of luteolin was relatively low and did not change under the influence of elicitors. On the other hand, in alfalfa sprouts the use of the Na-Sil increased luteolin glycosides almost 2-fold, but at the same time it decreased free luteolin content to the same extent. Orientin levels were very low in the sprouts of the legumes examined, and the elicitors used did not modify them.

*p*-Hydroxybenzoic acid (PHB) was quantitatively the main phenolic acid of fenugreek, lentils, and alfalfa sprouts (Table 4). Neither chlorogenic nor syringic acid were found in the sprouts of the tested legume species, while gallic acid was not analyzed due to technical difficulties. The use of Fe-EDTA–Na-Sil and Na-Sil during germination and growth of sprouts caused various changes in the content of free phenolic acids and their derivatives. The content of free PHB decreased under the influence of Na-Sil in lentil and alfalfa sprouts and increased in fenugreek. Moreover, Na-Sil caused an increase in the content of PHB esters in lentil and alfalfa sprouts and in glycosides in fenugreek sprouts. In turn, the content of PHB glycosides in lentil sprouts decreased after its influence. Ferulic acid in tested sprouts was mainly found as ester derivatives. In alfalfa sprouts, the elicitors used increased their content, while in lentil sprouts they caused a decrease. Both the used elicitors decreased the content of *p*-coumaric acid (PCA) esters in lentil sprouts and enhanced the content of PCA esters in alfalfa sprouts, while Na-Sil decreased the content of PCA esters in fenugreek sprouts. 

In addition to PHB, a relatively lower content of caffeic and sinapic acid were noted (Table 5). Esters and glycosides of caffeic and sinapic acids were present in smaller amounts than PHA and PCA. The used elicitors increased the content of caffeic acid esters in alfalfa sprouts but decreased their content in lentils. 

The total content of flavonoids in fenugreek sprouts was higher than that found for hydroxybenzoic or hydroxycinnamic acids (Table 6). In turn, the contents of phenolic acids and their derivatives in lentil and alfalfa sprouts were higher than those of flavonoids. The hydroxycinnamic acids were found mainly in the free and esterified forms but dominated the esterified phenolic fraction. The content of free flavonoids in fenugreek sprouts was markedly decreased under the influence of the applied elicitors, and at the same time their esters were found to be increased. However, the total contents of all flavonoids increased. A similar phenomenon occurred in alfalfa sprouts, in which, moreover, elicitors increased the total content of glycosides. In alfalfa sprouts, the effect of both elicitors was also due to the increase of total content of phenolic acids esters. Different results were obtained for lentil sprouts, in which the elicitors decreased the glycoside content of both flavonoids and phenolic acids, as well as the total content of all forms of phenolic acids and flavonoids. Generally, a greater effect of elicitors on flavonoid contents than on phenolic acids was observed (Table 6). 

## 3. Discussion

The content of phytochemicals in plants is dependent on stressful growth conditions [10,11,12,13]. In recent years, many reports have been published on the effects of various elicitors on sprout composition [29,30,31,32]. For example, it has been shown that soaking seeds in solutions containing iron compounds increased the concentration of this element in sprouts and the content of phenolic compounds [3,20,21]. Seeds soaked in Fe-EDTA and Na-Sil solutions germinate and grow under abiotic stress. It was previously found that silicates affected the antioxidant system by enhancing phenolics production in various plants [16]. Additionally, the presence of Fe during the germination of seeds led to increase the activity of antioxidative enzymes and content of phenolic compounds [20]. Furthermore, the use of iron chelate is beneficial because it can lead to the supplementation of sprout tissues with iron [3,21,22].

Application of potassium silicate increased the concentration of phenolic acids and flavonoids and enhanced the activity of enzymes in the phenylpropanoid pathway in response to infection by rose powdery mildew [53], but silicon had no effect on phenolics in plants in the absence of pathogen infection [54,55]. However, our previous studies showed that elicitation with Na-Sil and Fe-EDTA–Na-Sil significantly reduced the content of free (−)-epicatechin (EC) and quercetin but increased its esters and glycosides in common buckwheat sprouts [3]. An increase in EC ester content under the influence of the applied elicitors with a simultaneous decrease in free EC level also occurred in fenugreek and alfalfa sprouts. A particularly strong inhibitory effect on the accumulation of free EC was demonstrated under Na-Sil, which may indicate that the presence of iron chelate mitigated this phenomenon. However, both elicitors used increased the total flavonoid content in sprouts of both species, in contrast to buckwheat sprouts tested previously, in which the total flavonoid content decreased under the influence of these elicitors. EC content in lentil sprouts was much lower than in fenugreek and alfalfa, and the elicitors used did not affect its content. 

There is no information on the influence of the elicitors used in our study, Fe-EDTA and sodium metasilicate (Fe-EDTA–Na-Sil), on the composition of flavonoids and phenolic acids in legume sprouts. However, silicon application resulted in a decrease of phenylalanine ammonia lyase activity and a decline of phenolic content in cucumber roots [56]. The authors suggested that the decline in phenolics level was caused by the formation of Si complexes with phenol moiety. It is likely that the large decrease in free EC content under Na-Sil in fenugreek and alfalfa sprouts may be the result of the formation of silicon complexes with EC. However, this supposition needs to be confirmed in further studies by applying different doses of sodium metasilicate. 

Of the elicitors used, the mixture of iron chelate and sodium metasilicate had less effect on changes in flavonoid and phenolic acid content in legume sprouts than silicate alone. This may indicate a different effect of the two components. The observed phenomenon also occurred in a similar experiment with buckwheat sprouts [3]. Moreover, the application of iron chelate increased the content of this element in the sprouts of this species. This may provide an important rationale for recommending this elicitor to enrich sprouts with iron, which is deficient in people living in poor regions of the world [3].

The main difference is that in previously published papers EC was not found in the fenugreek, lentil, and alfalfa sprouts. In fenugreek sprouts, EC was found in more than 90% in the ester form, probably combined with gallic acid. Gallic acid was unable to be determined due to technical difficulties, although its presence in fenugreek sprouts and seeds was previously confirmed [57]. During routine sample analysis, an interference and/or high background in a multiple reaction monitoring (MRM) method for gallic acid was present, making it impossible to quantify this compound. In fenugreek sprouts of Moroccan origin, several phenolic compounds were detected: *C*-glycosides, flavonol *O*-diglycosides, flavone tri- and tetra *O*-, *C*-glycosides, and acylated flavone *O*-, *C*-glycosides, but not EC [58]. Among them, apigenin and luteolin derivatives were predominant [58]. A high content of EC is beneficial to health [43,44,45,46]. Its antioxidant activity is higher than that of common flavonols [59]. EC acts directly as an antioxidant, as well as indirectly, as it modulates the activity of antioxidant enzymes such as peroxide dismutase and glutathione peroxidase [60,61]. 

Our data indicate that the main flavonoids of lentil sprouts were glucosides of quercetin and kaempferol, while EC esters and apigenin glycosides were found in lower content. Earlier studies have shown quantitatively that in eight-day-old lentil sprouts the dominant flavonoid was catechin, and low content of naringenin, quercetin, and kaempferol have been noted [34]. In another study, kaempferol and quercetin glycosides acylated with sinapic and ferulic acid were found in lentil sprouts, and the main flavonoid was kaempferol-sinapoil-diglycoside [62]. On the other hand, a recently published paper showed that lentil sprouts contained mainly flavan-3-ols, but these were not identified [7]. The results of analyses of flavonoid content obtained in the present study for lentil sprouts are similar overall to the data obtained by Amarowicz et al. [63]. 

There are much less data on the composition and content of phenolic compounds in alfalfa sprouts. Earlier, it was found that alfalfa sprouts obtained in darkness contained glycosides of myricitin, morine, quercetin, and kaempferol [64]. In turn, data from another paper showed that alfalfa sprouts contain quercetin as the main flavonoid and low contents of myricetin, apigenin, and *iso*-rhamentin [65].

It is difficult to compare the results obtained for non-elicited legume sprouts with available literature data. This is due to many factors affecting the composition and content of metabolites and thus makes it difficult to compare the results obtained with those previously published. The large differences between the results of phenolic compounds in this study compared to previous reports may be mainly due to the different sprout production conditions. The sprouting time, presence, type, and intensity of light and the use or not of elicitors have a decisive influence on the composition and content of sprout metabolites [10,11,12,13,14,15,16,17]. The extraction procedure for isolation of the phenolic compounds could also have an impact on results of their analysis [48,49,50]. During our study for the extraction of phenolic compounds from freeze-dried sprout samples, a mixture of methanol, water, and formic acid was used. This type of extraction may be one of the factors that caused a difference in the polyphenolic profile in the examined sprouts compared to the results of other studies. Besides, other factors, such as genotype, environmental conditions during growth of the mother plant, and elicitors used can also affect the phytochemical contents in sprouts [2].

The results obtained for phenolic acid content in the legume sprouts partly support previously published data. According to these data, aqueous extracts of germinated fenugreek seeds contained gallic, caffeic, and *o*-coumaric and *p*-coumaric acid (PCA) [57,58,59]. In one-day-old, germinated fenugreek sprouts, caffeic acid and PCA and their derivatives were found [62]. Later, protocatechuic, caffeic, PCA, and ferulic and sinapic acids were detected in fenugreek seeds and sprouts [23], as well as gallic, caffeic, and syringic acids [62]. 

## 4. Material and Methods

### 4.1. Materials and Preparation of Sprouts

#### 4.1.1. Sprouting

Seeds of fenugreek (*Trigonella foenum-graecum* L.), lentil (*Lens culinaris* L.), and alfalfa (*Medicago sativa* L.) used for the study were purchased from Garden Seed and Nursery Stock Company Torseed Co., Torun, Poland. Initially, seeds without a seed coat were disinfected with 70% (*v*/*v*) ethanol for 1 min, followed with 2% sodium hypochlorite for 2 min, rinsed once in 0.01 N HCl, and 3 times with distilled water. The disinfected seeds were soaked at 24 °C in distilled water for 4 h and placed on a layer of sterilized and moist cotton gauze stretched over an open 330 mL jar. Over the next six days, the seeds and sprouts were soaked in distilled water (control) or elicitor solutions (Fe-EDTA–Na-Sil, Na-Sil). The soaking lasting 15 min was carried out twice each day, at 9:00 a.m. and 5:00 p.m. After each treatment, the seeds were placed back on the gauze layer. The sprouts were grown in a light conditions 100–120 µmol/(m^2^⋅s) photosynthetically active radiation (high pressure sodium lamps) at 20 ± 1 °C (day, 16 h) and 16 ± 2 °C (night, 8 h). On the seventh day, the obtained sprouts were collected and cut into 2–3 mm pieces and were freeze-dried in a laboratory freeze dryer (Alpha 1-2 LD plus, Martin Christ, Osterode am Harz, Germany) for 48 h and used for analyses of flavonoids and phenolic acids. 

#### 4.1.2. Elicitor Composition

The elicitors used in the study were solutions contained sodium metasilicate (Na_2_SiO_3_; Na-Sil, POCH, Poland), and a mixture of sodium metasilicate and iron chelate ((Fe-EDTA–Na-Sil), trademark Optysil, Intermag, Olkusz, Poland). Examined seeds or sprouts were soaked in distilled water (control), or in solutions containing sodium metasilicate with a concentration of 4 mM (Na-Sil), or with the Optysil, i.e., mixture of Fe-EDTA and sodium metasilicate, 0.5 mM Fe and 4 mM of Na_2_SiO_3_ (Fe-EDTA–Na-Sil), respectively. 

### 4.2. Analyses of Free and Derivatives of Flavonoids and Phenolic Acids 

Sprout samples were analyzed by HPLC–MS/MS for the determination of various forms of phenolic acids and flavonoids (free, esters, and glycosides). The profile and content of phenolic acids and flavonoids were determined according to the method of Płatosz et al. [66]. Briefly, a crude extract was obtained from freeze-dried plant samples by stirring overnight at 10 °C using a ThermoMixer (Benchmark Scientific, Saryeville, NJ, USA) with a mixture of methanol, water, and formic acid 80:19.9:0.1 (*v*/*v*/*v*). The extraction was repeated five times, and the obtained crude extracts were collected. Free forms of phenolic acids and flavonoids were isolated with diethyl after adjusting the initial extract to pH 2 with 6 M HCl. Next, after free forms isolation, esters present in the extracts were hydrolyzed in nitrogen atmosphere for 4 h at room temperature with 4 M NaOH. Subsequently, glycosides present in the extracts were hydrolyzed in the residues with 6 M HCl for 1 h at 100 °C. After each step, the released free forms were isolated with diethyl after adjusting the mixture to pH 2. The obtained ether extracts were evaporated to dryness under stream of nitrogen at 35 °C. The phenolics, both free and released form bound forms, were dissolved in 80% (*v*/*v*) methanol and centrifuged and subjected to HPLC–MS/MS analysis. Aliquots of extracts were injected into an HPLC system equipped with a HALO C18 column (2.7 μm particles, 0.5 × 50 mm, Eksigent, Vaughan, Canada) at 45 °C at a flow rate of 15 μL/min. The elution solvents were A (water/formic acid; 99.05/0.95; *v*/*v*) and B (acetonitrile/formic acid, 99.05/0.95, *v*/*v*). The gradient used was as follows: 5% B for 0.1 min, 5–90% B in 1.9 min, 90% B for 0.5 min, 90–5% B in 0.2 min, and 5% B for 0.3 min. For HPLC–MS/MS analysis, a QTRAP 5500 ion trap mass spectrometer (AB SCIEX, Vaughan, Canada) was applied. Optimal ESI-MS/MS conditions including nitrogen curtain gas (25 L/min), collision gas (9 L/min), ion spray source voltage (−4500 V), temperature (350 °C), nebulizer gas (35 L/min), and turbo gas (30 L/min) were applied.

Qualitative and quantitative analyses were conducted in the negative mode by multiple reaction monitoring (MRM) of selected ions in the first quadrupole and third quadrupole. The following flavonoids (free, esters, and glycosides) were determined: (‒)-epicatechin (EC), luteolin, orientin, vitexin, apigenin, naringenin, kaempferol, *iso*-rhamnetin, and quercetin. Moreover, derivatives (free, esters, glycosides) of the following phenolic acids were analyzed: 4-hydroxybenzoic (PHB), caffeic, sinapic, *p*-coumaric (PCA), ferulic, syringic, and chlorogenic. The recovery/extraction efficiency of phenolic compounds in the method used was at the level of 86.7 ± 1.1%–94.2 ± 0.9%, and the achieved results were not adjusted by the recovery factors. The external standards (0.01–0.50 g/mL) had linear calibration curves with a coefficient of determination of 0.997–0.999.

### 4.3. Statistics

Analyses of sprout tissues were performed in three replicates. Analysis of variance (one-way ANOVA) and Tukey’s post hoc test were used to check the significance of differences. Calculations were performed using Statistica 12PL software ((StatSoft, Tulsa, OK, USA). Results in tables are shown as mean ± standard deviation. Means marked with the same letter are statistically insignificant at *p* > 0.05 (post hoc Tukey’s test). Comparisons were made within each column—for each flavonoid and species separately. The term “not detected” in table descriptions means that the contents were below the method′s limit of determination (LOD).

## 5. Conclusions

The presence and high content of (−)-epicatechin (EC) in fenugreek sprouts was demonstrated for the first time. In the sprouts of elicited legumes, differential changes in the content of phenolic compounds were observed. However, it can be noted that the application of iron chelate and sodium silicate often caused a reduction in the content of free phenolic compounds in fenugreek and alfalfa sprouts. Such effects were found for EC, quercetin, apigenin, luteolin, and *p*-hydroxybenzoic acid, but this was accompanied by an increase in EC esters. Besides, in the alfalfa sprouts the levels of ferulic, *p*-hydroxycinnamic, *p*-hydroxybenzoic, and caffeic acid esters, and the glycosides of quercetin, apigenin, and luteolin increased. In turn, in the case of lentil sprouts, the elicitors reduced the contents of quercetin, kaempferol, caffeic acid, and *p*-hydroxybenzoic acid glycosides. In general, the used elicitors increased the contents of total phenolic compounds in fenugreek and alfalfa sprouts and decreased them in lentil sprouts. The use of sodium silicate alone increased the total phenolic compound content of fenugreek and alfalfa sprouts more than the mixture of silicate and iron chelate. Among the evaluated elicitors, Optysil seems to be worth recommending due to the presence of iron chelate, which can be used to enrich sprouts with this element.

## Figures and Tables

**Table 1:** The contents of epicatechin (EC) and naringenin (free and released from ester and *O*-glycoside forms) in legume sprouts (μg/g DW) treated with elicitors during growth.

Treatment	Free	Esters	Glycosides	Total
Epicatechin (EC)				
Fenugreek				
Control	2041 ± 27 ^a^	5045 ± 27 ^c^	nd	7086 ± 54 ^c^
Fe-EDTA–Na-Sil	1570 ±15 ^b^	6635 ± 44 ^b^	nd	8305 ± 59 ^a^
Na-Sil	319 ± 13 ^c^	8920 ± 48 ^a^	nd	9239 ± 61 ^a^
Lentil				
Control	nd	26.4 ± 2.0 ^a^	nd	26.4 ± 2.0 ^a^
Fe-EDTA–Na-Sil	nd	30.3 ± 2.1 ^a^	nd	30.3 ± 2.1 ^a^
Na-Sil	nd	35.3 ± 2.5 ^a^	nd	35.3 ± 2.5 ^a^
Alfalfa				
Control	112.5 ± 3.1 ^a^	316 ± 17 ^c^	nd	428 ± 20 ^c^
Fe-EDTA–Na-Sil	47.7 ± 2.0 ^b^	465 ± 24 ^b^	nd	513 ± 26 ^bc^
Na-Sil	34.2 ± 1.3 ^c^	626 ± 37 ^a^	nd	660 ± 38 ^ab^
Naringenin				
Fenugreek				
Control	1.4 ± 0.9 ^a^	1.4 ± 0.9 ^a^	6.3 ± 1.3 ^b^	9.6 ± 2.1 ^a^
Fe-EDTA–Na-Sil	1.5 ± 0.9 ^a^	1.0 ± 0.9 ^a^	16.8 ± 1.9 ^a^	5.3 ± 1.8 ^a^
Na-Sil	1.6 ± 0.9 ^a^	1.0 ± 0.9 ^a^	10.7 ± 1.4 ^ab^	3.5 ± 1.4 ^a^
Lentil				
Control	2.8 ± 1.0 ^a^	1.0 ± 1.0 ^a^	10.7 ± 1.3 ^a^	14.5 ± 3.3 ^a^
Fe-EDTA–Na-Sil	3.2 ± 1.0 ^a^	1.0 ± 0.9 ^a^	10.6 ± 1.2 ^a^	14.8 ± 3.1 ^a^
Na-Sil	7.5 ± 1.2 ^a^	1.9 ± 1.0 ^a^	17.2 ± 2.2 ^a^	26.6 ± 4.4 ^a^
Alfalfa				
Control	10.1 ± 1.4 ^b^	1.3 ± 1.0 ^a^	8.7 ± 1.0 ^a^	20.1 ± 3.4 ^a^
Fe-EDTA–Na-Sil	20.5 ± 1.8 ^a^	3.1 ± 1.0 ^a^	7.5 ± 1.0 ^a^	31.1 ± 3.8 ^a^
Na-Sil	12.6 ± 1.0 ^b^	1.0 ± 0.9 ^a^	16.4 ± 2.3 ^a^	30.0 ± 4.2 ^a^

Abbreviations: Fe-EDTA–Na-Sil—sodium metasilicate and Fe-EDTA chelate; Na-Sil—sodium metasilicate; nd—not detected; DW—dry weight. Mean results followed by the same letter within the same column, and calculated for each phenolic acid and species separately, were not significantly different (*p* < 0.05) according to Tukey’s test.

**Table 2:** The contents of quercetin and kaempferol (free and released from ester and *O*-glycoside forms) in legume sprouts (μg/g DW) treated with elicitors during growth.

Treatment	Free	Esters	Glycosides	Total
Quercetin				
Fenugreek				
Control	1.4 ± 0.9 ^a^	3.4 ± 1.2 ^a^	101 ± 5.1 ^a^	106 ± 7.2 ^a^
Fe-EDTA–Na-Sil	2.0 ± 1.0 ^a^	4.7 ± 1.0 ^a^	41.1 ± 5.2 ^b^	47.8 ± 7.2 ^b^
Na-Sil	2.0 ± 0.9 ^a^	3.7 ± 1.1 ^a^	63.5 ± 4.3 ^b^	69.2 ± 6.3 ^b^
Lentil				
Control	3.0 ± 0.9 ^a^	nd	345 ± 5.9 ^a^	348 ± 6.8 ^a^
Fe-EDTA–Na-Sil	3.3 ± 1.3 ^a^	nd	153 ± 3.7 ^b^	156 ± 5.0 ^b^
Na-Sil	3.9 ± 1.2 ^a^	nd	8.4 ± 1.9 ^c^	12.3 ± 3.1 ^c^
Alfalfa				
Control	31.8 ± 1.3 ^a^	58.3 ± 3.5 ^a^	165 ± 3.9 ^b^	255 ± 8.7 ^b^
Fe-EDTA–Na-Sil	35.3 ± 2.1 ^a^	37.4 ± 2.7 ^b^	177 ±2.6 ^b^	250 ± 6.4 ^b^
Na-Sil	23.0 ± 1.0 ^b^	42.8 ± 2.5 ^b^	344 ± 6.7 ^a^	410 ± 10.2 ^a^
Kaempferol				
Fenugreek				
Control	nd	nd	32.5 ± 2.2 ^a^	32.5 ± 2.2 ^a^
Fe-EDTA–Na-Sil	nd	nd	30.9 ± 1.2 ^a^	30.9 ± 1.2 ^a^
Na-Sil	nd	nd	31.3 ± 2.4 ^a^	31.3 ± 2.4 ^a^
Lentil				
Control	nd	3.3 ± 0.9 ^a^	122 ± 3.9 ^b^	125 ± 4.8 ^b^
Fe-EDTA–Na-Sil	nd	3.5 ± 1.1 ^a^	157 ± 4.9 ^a^	160 ± 6.0 ^a^
Na-Sil	nd	4.7 ± 1.0 ^a^	98.7 ± 2.8 ^c^	102 ± 3.8 ^b^
Alfalfa				
Control	3.6 ± 0.9 ^a^	1.0 ± 0.9 ^a^	29.1 ± 1.9 ^c^	33.7 ± 3.7 ^b^
Fe-EDTA–Na-Sil	5.4 ± 1.2 ^a^	1.2 ± 0.9 ^a^	45.2 ± 3.5 ^b^	51.8 ± 5.6 ^ab^
Na-Sil	1.5 ± 0.9 ^a^	1.7 ± 0.9 ^a^	71.2 ± 4.4 ^a^	74.4 ± 6.2 ^a^

Abbreviations: Fe-EDTA–Na-Sil—sodium metasilicate and Fe-EDTA chelate; Na-Sil—sodium metasilicate; nd—not detected; DW—dry weight. Mean results followed by the same letter within the same column, and calculated for each phenolic acid and species separately, were not significantly different (*p* < 0.05) according to Tukey’s test.

**Table 3:** The contents of flavones (free and released from ester and *O*-glycoside forms) in legume sprouts (μg/g DW) treated with elicitors during growth.

Treatment	Free	Esters	Glycosides	Total
Apigenin				
Fenugreek				
Control	1.9 ± 0.4 ^a^	3.3 ± 0.8 ^a^	95.1 ± 1.4 ^a^	100 ± 2.6 ^a^
Fe-EDTA–Na-Sil	1.7 ± 0.4 ^a^	3.8 ± 0.4 ^a^	93.5 ± 1.8 ^a^	99.0 ± 2.8 ^a^
Na-Sil	3.7 ± 0.8 ^a^	4.2 ± 0.3 ^a^	95.3 ± 1.2 ^a^	103 ± 2.4 ^a^
Lentil				
Control	nd	nd	nd	nd
Fe-EDTA–Na-Sil	nd	nd	nd	nd
Na-Sil	nd	nd	nd	nd
Alfalfa				
Control	238 ± 2.8 ^a^	4.7 ± 0.7 ^a^	252 ± 2.9 ^c^	495 ± 6.4 ^c^
Fe-EDTA–Na-Sil	181 ± 2.4 ^b^	5.4 ± 0.9 ^a^	345 ± 2.8 ^b^	531 ± 5.1 ^b^
Na-Sil	105 ± 1.6 ^c^	6.8 ± 0.7 ^a^	535 ± 4.8 ^a^	646 ± 7.1 ^a^
Vitexin (apigenin-8-*C*-glucoside)				
Fenugreek				
Control	nd	10.5 ± 1.0 ^a^	nd	10.5 ± 1.0 ^a^
Fe-EDTA–Na-Sil	nd	9.8 ± 1.2 ^a^	nd	9.8 ± 1.2 ^a^
Na-Sil	nd	5.2 ± 1.0 ^a^	nd	5.2 ± 1.0 ^a^
Lentil				
Control	nd	nd	nd	nd
Fe-EDTA–Na-Sil	nd	nd	nd	nd
Na-Sil	nd	nd	nd	nd
Alfalfa				
Control	nd	nd	nd	nd
Fe-EDTA–Na-Sil	nd	nd	nd	nd
Na-Sil	nd	nd	nd	nd
Luteolin				
Fenugreek				
Control	1.7 ± 1.2 ^a^	nd	28.1 ± 2.7 ^a^	29.8 ± 3.9 ^a^
Fe-EDTA–Na-Sil	1.3 ± 1.2 ^a^	nd	17.5 ± 1.9 ^a^	18.8 ± 3.1 ^a^
Na-Sil	1.1± 1.1 ^a^	nd	19.1 ± 2.2 ^a^	20.2 ± 3.3 ^a^
Lentil				
Control	5.4 ± 1.0 ^a^	nd	29.9 ± 2.3 ^a^	35.3 ± 3.3 ^a^
Fe-EDTA–Na-Sil	5.8 ± 1.1 ^a^	nd	28.3 ± 1.5 ^a^	34.1 ± 2.6 ^a^
Na-Sil	5.8 ± 1.2 ^a^	nd	20.1 ± 1.9 ^a^	25.9 ± 3.1 ^a^
Alfalfa				
Control	50.2 ± 3.5 ^a^	7.8 ± 1.5 ^a^	27.4 ± 2.9 ^b^	85.4 ± 7.9 ^a^
Fe-EDTA–Na-Sil	62.9 ± 5.5 ^a^	7.5 ± 1.0 ^a^	31.6 ± 3.1 ^b^	102 ± 9.6 ^a^
Na-Sil	20.9 ± 2.2 ^b^	2.8 ± 1.1 ^a^	58.6 ± 4.7 ^a^	82.3 ± 8.0 ^a^
Orientin (luteolin-8-C-glucoside)				
Fenugreek				
Control	nd	9.6 ± 2.1 ^a^	nd	9.6 ± 2.1 ^a^
Fe-EDTA–Na-Sil	nd	5.3 ± 1.8 ^a^	nd	5.3 ± 1.8 ^a^
Na-Sil	nd	3.5 ± 1.4 ^a^	nd	3.5 ± 1.4 ^a^
Lentil				
Control	nd	nd	nd	nd
Fe-EDTA–Na-Sil	nd	nd	nd	nd
Na-Sil	nd	nd	nd	nd
Alfalfa				
Control	nd	nd	nd	nd
Fe-EDTA–Na-Sil	nd	nd	nd	nd
Na-Sil	nd	nd	nd	nd

Abbreviations: Fe-EDTA–Na-Sil—sodium metasilicate and Fe-EDTA chelate; Na-Sil—sodium metasilicate; nd—not detected; DW—dry weight. Mean results followed by the same letter within the same column, and calculated for each phenolic acid and species separately, were not significantly different (*p* < 0.05) according to Tukey’s test.

**Table 4:** The contents of major phenolic acids (free and released from ester and *O*-glycoside forms) in legume sprouts (μg g^−1^ DW) treated with elicitors during growth.

Treatment	Free	Esters	Glycosides	Total
*p*-hydroxybenzoic acid (PHB)				
Fenugreek				
Control	217 ± 1.2 ^c^	265 ± 4.4 ^a^	1129 ± 4.2 ^b^	1611 ± 9.8 ^b^
Fe-EDTA–Na-Sil	233 ± 3.5 ^b^	276 ± 2.0 ^a^	1071 ± 7.3 ^c^	1580 ± 12.8 ^b^
Na-Sil	334 ± 3.4 ^a^	273 ± 1.5 ^a^	1434 ± 4.5 ^a^	2041 ± 9.4 ^a^
Lentil				
Control	275 ± 2.8 ^b^	207 ± 1.0 ^b^	1045 ± 4.1 ^a^	1527 ± 7.9 ^a^
Fe-EDTA–Na-Sil	304 ± 2.6 ^a^	226 ± 8.1 ^b^	793 ± 6.0 ^b^	1491 ± 17.7 ^a^
Na-Sil	246 ± 3.1 ^c^	327 ± 3.0 ^a^	824 ± 6.0 ^b^	1264 ± 12.1 ^b^
Alfalfa				
Control	264 ± 2.3 ^a^	188 ± 0.9 ^c^	749 ± 7.5 ^b^	1200 ± 10.7 ^c^
Fe-EDTA–Na-Sil	276 ± 2.6 ^a^	329 ± 3.3 ^a^	886 ± 2.0 ^a^	1491 ± 7.9 ^a^
Na-Sil	214 ± 3.0 ^b^	274 ± 3.0 ^b^	776 ± 6.0 ^b^	1264 ± 12.0 ^b^
Ferulic acid				
Fenugreek				
Control	2.4 ± 0.3 ^a^	14.1 ± 1.0 ^a^	3.3 ± 0.2 ^a^	19.8 ± 1.5 ^a^
Fe-EDTA–Na-Sil	2.6 ± 0.7 ^a^	12.7 ± 1.2 ^a^	4.8 ± 0.9 ^a^	20.l ± 2.8 ^a^
Na-Sil	3.2 ± 0.6 ^a^	12.4 ± 1.1 ^a^	5.1 ± 0.7 ^a^	20.7 ± 2.4 ^b^
Lentil				
Control	3.0 ± 0.9 ^a^	237 ± 4.4 ^a^	4.3 ± 0.7 ^a^	244 ± 5.0 ^a^
Fe-EDTA–Na-Sil	2.3 ± 0.5 ^a^	218 ± 8.0 ^ab^	4.2 ± 0.5 ^a^	225 ± 9.0 ^b^
Na-Sil	1.6 ± 0.7 ^a^	148 ± 4.0 ^b^	3.1 ± 0.6 ^a^	152 ± 5.3 ^c^
Alfalfa				
Control	3.0 ± 0.9 ^a^	438 ± 4.9 ^c^	3.6 ± 0.7 ^a^	449 ± 5.5 ^c^
Fe-EDTA–Na-Sil	5.9 ± 0.5 ^a^	594 ± 9.0 ^a^	3.4 ± 0.5 ^a^	604 ± 10.0 ^a^
Na-Sil	5.0 ± 0.7 ^a^	533 ± 4.3 ^b^	5.5 ± 0.6 ^a^	543 ± 5.6 ^b^
*p*-coumaric acid (PCA)				
Fenugreek				
Control	5.1 ± 1.3 ^a^	117 ± 3.7 ^a^	10.3 ± 1.1 ^a^	132 ± 6.1 ^a^
Fe-EDTA–Na-Sil	10.7 ± 1.7 ^a^	104 ± 2.2 ^a^	12.6 ± 0.9 ^a^	127 ± 4.8 ^a^
Na-Sil	10.1 ± 1.6 ^a^	63.0 ± 1.7 ^b^	11.4 ± 1.2 ^a^	84.5 ± 4.5 ^b^
Lentil				
Control	5.5 ± 0.3 ^a^	432 ± 4.9 ^a^	7.3 ± 0.7 ^a^	445 ± 5.9 ^a^
Fe-EDTA–Na-Sil	3.3 ± 0.5 ^a^	365 ± 12.0 ^b^	4.3 ± 0.5 ^b^	372 ± 13.0 ^b^
Na-Sil	3.1 ± 0.7 ^a^	267 ± 4.3 ^c^	9.0 ± 0.6 ^a^	279 ± 5.6 ^c^
Alfalfa				
Control	5.6 ± 0.7 ^a^	416 ± 1.9 ^c^	4.8 ± 0.7 ^a^	426 ± 3.3 ^c^
Fe-EDTA–Na-Sil	7.8 ± 1.1 ^a^	565 ± 2.8 ^b^	7.1 ± 0.6 ^a^	580 ± 4.5 ^b^
Na-Sil	5.4 ± 1.0 ^a^	714 ± 3.0 ^a^	6.6 ± 0.7 ^a^	725 ± 4.7 ^a^

Abbreviations: Fe-EDTA–Na-Sil—sodium metasilicate and Fe-EDTA chelate; Na-Sil—sodium metasilicate; nd—not detected; DW—dry weight. Mean results followed by the same letter within the same column, and calculated for each phenolic acid and species separately, were not significantly different (*p* < 0.05) according to Tukey’s test.

**Table 5:** The contents of minor phenolic acids (free and released from ester and *O*-glycoside forms) in legume sprouts (μg/g^−1^ DW) treated with elicitors during growth.

Treatment	Free	Esters	Glycosides	Total
Caffeic acid				
Fenugreek				
Control	5.9 ± 1.0 ^a^	9.6 ± 0.7 ^a^	10.4 ± 1.1 ^a^	25.9 ± 2.8 ^b^
Fe-EDTA–Na-Sil	4.6 ± 0.9 ^a^	7.8 ± 1.0 ^a^	9.4 ± 1.2 ^a^	21.8 ± 3.1 ^b^
Na-Sil	6.3 ± 0.5 ^a^	17.7 ± 3.1 ^a^	12.6 ± 1.0^a^	36.6 ± 2.5 ^a^
Lentil				
Control	4.1	102.6 ± 5.1 ^a^	14.6 ± 1.1 ^a^	121.3 ± 6.2 ^a^
Fe-EDTA–Na-Sil	nd	20.9 ± 2.0 ^b^	9.5 ± 1.0 ^ab^	30.4 ± 3.0 ^b^
Na-Sil	nd	6.8 ± 1.0 ^c^	7.3 ± 0.9 ^b^	14.1 ± 1.9 ^c^
Alfalfa				
Control	nd	93.7 ± 0.9 ^c^	9.7 ± 1.5 ^a^	103 ± 2.4 ^c^
Fe-EDTA–Na-Sil	nd	164 ± 3.3 ^b^	10.3 ± 2.0 ^a^	175 ± 5.3 ^b^
Na-Sil	nd	262 ± 3.0 ^a^	12.4± 2.0 ^a^	274 ± 5.0 ^a^
Sinapic acid				
Fenugreek				
Control	nd	7.1 ± 0.7 ^a^	1.0 ± 0.4 ^a^	8.1 ± 1.1 ^a^
Fe-EDTA–Na-Sil	nd	4.6 ± 1.2 ^a^	1.2 ± 0.5 ^a^	5.8 ± 1.7 ^a^
Na-Sil	1.1 ± 0.4	5.8 ± 1.0 ^a^	1.4 ± 0.2 ^a^	8.3 ± 1.6 ^a^
Lentil				
Control	nd	3.7 ± 0.9 ^a^	1.6 ± 0.3 ^a^	5.3 ± 1.2 ^a^
Fe-EDTA–Na-Sil	nd	3.4 ± 0.5 ^a^	1.4 ± 0.3 ^b^	4.8 ± 0.8 ^a^
Na-Sil	nd	1.3 ± 0.7 ^a^	1.3 ± 0.2 ^a^	2.6 ± 0.9 ^a^
Alfalfa				
Control	nd	3.9± 0.9 ^b^	2.0 ± 0.7 ^a^	5.9 ± 1.3 ^a^
Fe-EDTA–Na-Sil	nd	6.7 ± 0.8 ^ab^	2.4 ± 0.6 ^a^	9.1 ± 1.4 ^a^
Na-Sil	nd	9.5 ± 1.0 ^a^	2.2 ± 0.7 ^a^	11.7 ± 1.7 ^a^

Abbreviations: Fe-EDTA–Na-Sil—sodium metasilicate and Fe-EDTA chelate; Na-Sil—sodium metasilicate; nd—not detected; DW—dry weight. Mean results followed by the same letter within the same column, and calculated for each phenolic acid and species separately, were not significantly different (*p* < 0.05) according to Tukey’s test.

**Table 6:** The contents of total flavonoids and phenolic acids (free and released from ester and *O*-glycoside forms) in legume sprouts (μg/g DW) treated with elicitors during growth.

Treatment	Free	Esters	Glycosides	Total
Flavonoids				
Fenugreek				
Control	2047	5075	263	7385
Fe-EDTA–Na-Sil	1576	6661	198	8435
Na-Sil	327	8938	218	9483
Lentil				
Control	27.1	30.9	513	571
Fe-EDTA–Na-Sil	39.7	32.4	357	429
Na-Sil	50.5	46.0	148	245
Alfalfa				
Control	446	389	464	1299
Fe-EDTA–Na-Sil	352	520	606	1478
Na-Sil	197	681	1025	1903
Phenolic acids				
Fenugreek				
Control	230	413	1154	1797
Fe-EDTA–Na-Sil	252	405	1099	1756
Na-Sil	355	372	1464	2191
Lentil				
Control	287	982	1073	2342
Fe-EDTA–Na-Sil	309	834	812	1955
Na-Sil	251	750	844	1845
Alfalfa				
Control	276	1140	768	2184
Fe-EDTA–Na-Sil	290	1660	909	2859
Na-Sil	225	1792	802	2819

Abbreviations: Fe-EDTA–Na-Sil—sodium metasilicate and Fe-EDTA chelate; Na-Sil—sodium metasilicate; DW—dry weight.

## Data Availability

The data presented in this study are available in this article

## Sample Availability

Not available.
